# Low level laser therapy promotes bone regeneration by coupling angiogenesis and osteogenesis

**DOI:** 10.1186/s13287-021-02493-5

**Published:** 2021-08-03

**Authors:** Jie Bai, Lijun Li, Ni Kou, Yuwen Bai, Yaoyang Zhang, Yun Lu, Lu Gao, Fu Wang

**Affiliations:** 1 School of Stomatology, Dalian Medical University, Dalian, 116044 China; 2 The Affiliated Stomatological Hospital of Dalian Medical University School of Stomatology, Dalian, 116027 China; 3 Academician Laboratory of Immune and Oral Development & Regeneration, Dalian Medical University, Dalian, 116044 China

**Keywords:** Low level laser therapy, Type H vessels, Angiogenesis, Osteogenesis, Reactive oxygen species, Hypoxia-inducible factor 1α

## Abstract

**Background:**

Bone tissue engineering is a new concept bringing hope for the repair of large bone defects, which remains a major clinical challenge. The formation of vascularized bone is key for bone tissue engineering. Growth of specialized blood vessels termed type H is associated with bone formation. In vivo and in vitro studies have shown that low level laser therapy (LLLT) promotes angiogenesis, fracture healing, and osteogenic differentiation of stem cells by increasing reactive oxygen species (ROS). However, whether LLLT can couple angiogenesis and osteogenesis, and the underlying mechanisms during bone formation, remains largely unknown.

**Methods:**

Mouse bone marrow mesenchymal stem cells (BMSCs) combined with biphasic calcium phosphate (BCP) grafts were implanted into C57BL/6 mice to evaluate the effects of LLLT on the specialized vessel subtypes and bone regeneration in vivo. Furthermore, human BMSCs and human umbilical vein endothelial cells (HUVECs) were co-cultured in vitro. The effects of LLLT on cell proliferation, angiogenesis, and osteogenesis were assessed.

**Results:**

LLLT promoted the formation of blood vessels, collagen fibers, and bone tissue and also increased CD31^hi^EMCN^hi^-expressing type H vessels in mBMSC/BCP grafts implanted in mice. LLLT significantly increased both osteogenesis and angiogenesis, as well as related gene expression (HIF-1α, VEGF, TGF-β) of grafts in vivo and of co-cultured BMSCs/HUVECs in vitro. An increase or decrease of ROS induced by H_2_O_2_ or Vitamin C, respectively, resulted in an increase or decrease of HIF-1α, and a subsequent increase and decrease of VEGF and TGF-β in the co-culture system. The ROS accumulation induced by LLLT in the co-culture system was significantly decreased when HIF-1α was inhibited with DMBPA and was followed by decreased expression of VEGF and TGF-β.

**Conclusions:**

LLLT enhanced vascularized bone regeneration by coupling angiogenesis and osteogenesis. ROS/HIF-1α was necessary for these effects of LLLT. LLLT triggered a ROS-dependent increase of HIF-1α, VEGF, and TGF-β and resulted in subsequent formation of type H vessels and osteogenic differentiation of mesenchymal stem cells. As ROS also was a target of HIF-1α, there may be a positive feedback loop between ROS and HIF-1α, which further amplified HIF-1α induction via the LLLT-mediated ROS increase. This study provided new insight into the effects of LLLT on vascularization and bone regeneration in bone tissue engineering.

**Supplementary Information:**

The online version contains supplementary material available at 10.1186/s13287-021-02493-5.

## Introduction

Bone defects caused by trauma, surgery, tumor, congenital disease, and other pathological factors impair both structure and function, seriously affecting the quality of life and physical and mental health of patients. At present, treatment of bone defects mainly includes autologous and allogeneic bone transplantation, or biomaterial filling [1–4]. With the development of biotechnology and progress in regenerative medicine, tissue engineering technology has shown great potential in repairing bone defects. Tissue engineering has the advantages of unlimited tissue sources and low antigenicity, which avoids the donor site injury of autologous bone grafting and the recipient immune rejection caused by allogeneic bone transplantation [5, 6]. For successful tissue-engineered bone repair and regeneration, it is essential to restore the nutrition supply and promote osteogenic differentiation of stem cells as early as possible.

Adequate bone blood flow plays an important role in bone regeneration [7]. For the repair of large bone defects, the speed of vascularization of bone tissue-engineered grafts is often slow due to insufficient blood supply at the initial stage, resulting in graft ischemia and necrosis [8]. The physiological process of long bone development involves bone modeling and bone remodeling, both of which are coupled with angiogenesis [9]. In bone modeling associated with endochondral ossification, hypertrophic chondrocytes express high levels of vascular endothelial growth factor (VEGF), which promotes the vascular invasion of cartilage and recruits chondroclasts to resorb hypertrophic cartilage and osteoblasts to build the bone matrix [7, 10]. Kusumbe et al. proposed the terminology for bone microvessels: type H for the high expression of CD31 and Endomucin (CD31^hi^EMCN^hi^) subset and type L for the weak expression CD31 and EMCN (CD31^lo^/EMCN^lo^) sinusoidal vessels [11]. Type H was identified as a special subtype of blood vessels in trabecular and cortical bone adjacent to the growth plate and along the periosteal and endosteal surface, respectively, which angiogenesis is tightly coupled with osteogenesis, suggesting the existence of molecular communications between endothelial cells (ECs) and osteoblasts [12]. Targeting type H vessels in osteogenesis will provide a potential therapeutic approach for various bone disorders.

Hypoxia-inducible factor 1α (HIF-1α) is a transcription factor directly regulated by hypoxia [13]. Osteoblasts and nearby ECs may increase HIF-1α expression during osteogenesis. HIF-1α activity upregulates VEGF expression in hypoxic tissues, making HIF-1α essential for wound regeneration and tumor vascularization [14, 15]. Studies demonstrated that endothelial HIF-1α is a significant promoter of type H vessel formation in the metaphysis. Specific deletion of HIF-1α in endothelial cells resulted in a significant reduction of osteoprogenitors, associated with a decrease in trabecular bone formation [12]. Therefore, HIF-1α signaling plays a vital role in the regulation of type H vessel abundance and couples angiogenesis with osteogenesis.

Low level laser therapy (LLLT), also known as photobiomodulation (PBM), is a treatment that uses low level lasers or light-emitting diodes (LEDs) to modulate cell functions. LLLT can regulate cell functions by affecting cell growth and secretion of cytokines, thereby exerting a variety of biological effects [16–18]. Many studies have shown that LLLT has positive photobiostimulatory effects on cell proliferation, ECs angiogenesis, osteogenic differentiation, bone regeneration, and fracture healing [19–21]. Accumulating evidence has indicated that cells exposed to laser irradiation undergo a sustained increase in the production of endogenous reactive oxygen species (ROS) through changing mitochondrial membrane potential. ROS can promote the oxidation of ferrous ions (Fe2 +) and inhibit the activation of proline hydroxylases (PHDs), thereby inhibition of HIF-1α degradation [22, 23]. Thus, the ROS/HIF-1α signal pathway plays a critical role in the osteogenic progression induced by LLLT treatment. In addition, the transforming growth factor-β (TGF-β) superfamily is encoded by 23 different genes, which have been demonstrated to form complex interactions and dependence in regulating stem cell pluripotency and differentiation in various experimental models. Previous studies reported that LLLT could activate latent TGF-β by inducing ROS in a dose-dependent manner, and TGF-β has the ability to induce human dental stem cells differentiate into odontoblast cells in vitro [24, 25].

LLLT plays an important role in promoting osteogenic differentiation of stem cells and angiogenesis. Here, we propose a hypothesis that LLLT may enhance bone repair and regeneration by coupling angiogenesis and osteogenesis. Our in vivo experiments showed that LLLT promoted implanted BCP or mBMSCs/BCP to form more bone tissue and type H blood vessels with a high expression of cytokines related to angiogenesis and osteogenesis. We then discovered that LLLT promoted the crosstalk between ECs and mesenchymal stem cells (MSCs) using an in vitro co-culture system. Our results suggested that LLLT triggered a ROS-dependent increase of HIF-1α and TGF-β, which resulted in subsequent crosstalk between angiogenesis and osteogenesis. Our results provided a scientific basis for the effect of LLLT on the coupling of vascularization and bone regeneration and will improve the success of clinical application of bone tissue engineering techniques.

## Materials and methods

### Isolation and culture of cells

For human studies, approval from the Ethics Committee of Dalian Medical University as well as written informed consent from all participants was obtained. The study was performed in accordance with the principles of the Declaration of Helsinki. Human bone marrow mesenchymal stem cells (hBMSCs) were isolated from jawbone removed from patients undergoing orthognathic surgery at the Affiliated Stomatological Hospital of Dalian Medical University. The hBMSCs were cultured and expanded following the established method [26], in low glucose Dulbecco’s Modified Eagle’s Medium (L-DMEM, Hyclone, Logan, UT, USA), containing 1% penicillin–streptomycin (Gibco; Thermo Fisher Scientific, Inc., Waltham, MA, USA) and 10% fetal bovine serum (FBS; Gibco, Thermo Fisher Scientific, Inc.) with 5% CO_2_ at 37°C. hBMSCs up to passage 5 were used in this study.

The isolation of mouse bone marrow mesenchymal stem cells (mBMSCs) was performed as described previously [27]. The mBMSCs were cultured in MEM alpha modification (α-MEM, Hyclone) supplemented with 10% FBS and 1% penicillin-streptomycin. The mBMSCs at passages 3–5 were used for the in vivo study. Human umbilical vein endothelial cells (HUVECs; American Type Culture Collection, ATCC, Manassas, VA, USA) were cultured in endothelial cell medium (ECM, ScienCell, San Diego, CA, USA) with 5% FBS, 1% penicillin-streptomycin, and 1% endothelial cell growth supplement (ECGS, ScienCell) at 37°C with 5% CO_2_.

### Osteogenic differentiation and assay

The cells were divided into three groups: HUVECs, hBMSCs, and co-culture (hBMSCs and HUVECs co-cultured at 5:1) in 24-well plates (5 × 10^4^ cells/well). When the cells reached 100% confluence, the medium was changed to osteogenic medium that comprised α-MEM (Hyclone), supplemented with 10% FBS (Gibco), 10^−7^ M dexamethasone (Sigma-Aldrich, St. Louis, MO, USA), 10 mM β-glycerophosphate disodium (Coolaber, Beijing, China), and 50 μg/ml L-ascorbic acid (Coolaber). Alkaline phosphatase (ALP) staining, Alizarin Red (ARS) staining, and semi-quantitative analysis were conducted according to the manufacturer’s instructions. To evaluate the ALP, five fields of view were randomly selected under 10× magnification, and then, the images were quantified in the average of optical density by the Image-Pro Plus 7.0 version software (Media Cybernetics, Inc., Rockville, MD, USA). For ARS, the alizarin red was eluted with 10 % cetylpyridinium chloride (CPC, Sigma-Aldrich), and the absorbance was measured at 570nm using a microplate reader (Molecular Devices, Sunnyvale, CA, USA).

### Construction of the mBMSCs/BCP complex and the surgical procedure

Biphasic calcium phosphate (BCP), purchased from the Biomaterial Engineering Research Center of Sichuan University (Chengdu, China), was composed of 60% hydroxyapatite (HA) and 40% β-tricalcium phosphate (β-TCP). A porous cylinder of BCP was custom made with a 3-mm diameter× 2-mm height. The porosity was approximately 50%, and the pore size ranged from 300 to 500 μM. To create the mBMSC/BCP complex, mBMSCs (2 × 10^6^/mL) were seeded into BCP sterilized by irradiation and incubated for 3 h for in vivo implantation, or cultured for 7 days for in vitro tests.

Five-week-old C57BL/6 female mice were provided by the Experimental Animal Center of Dalian Medical University. All the experiments were approved by the Institutional Animal Care and Use Committee of Dalian Medical University. A total of 40 mice were randomly divided into four groups: BCP group, BCP alone without LLLT; BCP+LLLT group, the implanted BCP received LLLT every other day for 3 weeks (from 7 days prior to surgery to 14 days post-operation); BCP+mBMSCs group, mBMSCs/BCP without LLLT; and BCP+mBMSCs+LLLT group, the implanted mBMSCs/BCP received LLLT every other day for 3 weeks (from 7 days prior to surgery to 14 days post-operation). For graft implantation, a transverse incision was made at the midpoint of outer canthus line in the mouse. The mucoperiosteal was stripped to the herringbone suture, and the BCP or mBMSCs/BCP was implanted under the calvarial periosteum of the mice at the midpoint of the herringbone suture (posterior fontanel) under anesthesia, and the incision was sutured.

### LLLT protocol

A low energy laser apparatus (GaAlAs semiconductor laser JLT-MD500B, Jinlaite Optoelectronics, Co., Ltd., Wuhan, China) with a continuous wavelength of 808 nm was used in this study. The total energy density of the irradiation spot was measured using a laser power meter. For in vitro LLLT, the cells were irradiated with 4.5 J/cm^2^ (distance 14 cm, power 100 mW, time 3 min) every day. For in vivo LLLT, the implantation area of the anesthetized mice received LLLT (irradiation dose 1.8 J/cm^2^, 808 nm; distance 14 cm; power 40 mW; time 3 min; irradiation area 4 cm^2^). At 1 or 2 months after operation, the implanted grafts were isolated for histological staining.

### Histological staining

Hematoxylin and eosin (H&E) and Masson’s trichrome staining (Solarbio, Co., Ltd., Beijing, China) were performed according to the manufacturer’s instructions.

For immunofluorescence staining, the sections were incubated using the primary antibodies (rabbit polyclonal anti-CD31 antibodies, 1:10, ab28364, Abcam; rat monoclonal anti-EMCN antibodies, 1:10, ab106100; Abcam, Cambridge, MA, USA) for 24 h at 4°C. Then the secondary goat anti-rabbit antibodies conjugated with Cy3 568 (1:200 diluted in PBS, ab97075; Abcam) and goat anti-rat conjugated with dylight 488 (1:200 diluted in PBS, A23240; Abbkine, Wuhan, China) were used to incubate the tissues for 1 h at 37°C. Finally, the nuclei were counterstained with 4′,6-diamidine-2′-phenylindole dihydrochloride (DAPI, 1:1,000 diluted in PBS, 10236276001; Roche, Basel, Switzerland). The images were photographed under a fluorescence microscope (Olympus Corporation, Tokyo, Japan) and evaluated using Image-Pro Plus version 7.0 software (Media Cybernetics, Inc., Rockville, MD, USA).

For immunohistochemistry, the sections were incubated with primary antibodies (rabbit polyclonal anti-HIF-1α antibodies, 1:400, ab2185, Abcam; rabbit polyclonal anti-TGF-β, 1:200, ab31013, Abcam; mouse monoclonal anti-VEGF, 1:500, ab1316; Abcam) overnight at 4°C, then with the horseradish peroxidase (HRP)-conjugated goat anti-rabbit streptavidin (ZSGB-BIO, Beijing, China) secondary antibody at room temperature for 2 h. Finally, the sections were visualized using 3,3-diaminobenzidine (DAB; ZSGB-BIO), and counterstained with hematoxylin (Solarbio, Beijing, China).

### Cell proliferation assay

The effect of LLLT on cell proliferation was assessed by Cell Counting Kit-8 (CCK8, ApexBio Technology, Inc., Houston, TX, USA). According to the manufacturer’s instructions, cells were seeded in a 96-well plate at a density of 2×10^3^ cells / well in 100 μL of culture medium with LLLT (0 J/cm^2^, 1.5 J/cm^2^, 4.5 J/cm^2^, 7.5 J/cm^2^) for 1-5 days. CCK-8 solution of 10 μL was added to each well of the plate, and then the plate was incubated for 1 hour in the incubator at 37°. Finally, the absorbance was measured at 490 nm using a microplate reader.

### Tube formation assay

The cells were seeded into 96-well plates precoated with BD Matrigel™ Matrix (BD Biosciences, Franklin Lakes, NJ, USA) and incubated for 3 h at 37°C. The formed capillary-like structures were fixed and observed under a light microscope (Olympus Corporation). Tube formation was quantified using ImageJ software.

### Quantitative real-time polymerase chain reaction (RT-qPCR)

RT-qPCR was performed to evaluate the mRNA expression levels of osteogenesis and angiogenesis genes in HUVECs, hBMSCs, and co-cultured cells. Total RNA was isolated with RNAiso Plus (TRIzol; Takara Bio, Inc., Otsu, Japan) and reverse transcribed to cDNA with HiScript II Q RT SuperMix (Vazyme Biotech, Co., Ltd., Nanjing, China). RT-qPCR was performed using ChamQ Universal SYBR qPCR Master Mix (Vazyme Biotech). GAPDH was used as the internal control for each experiment. The primer sequences for RT-qPCR are listed in Additional file 1: Table S1. Data were analyzed using the 2−ΔΔCt relative expression method.

### Immunoblotting

Protein was isolated from cells in radio immunoprecipitation assay lysis buffer (RIPA, Solarbio) and quantified using a bicinchoninic acid protein assay kit (BCA; Beyotime, Shanghai, China). Then the protein was loaded onto a polyacrylamide gel and electroblotted onto polyvinylidene difluoride (PVDF) membranes (Millipore, Merck KGaA, Darmstadt, Germany). The membrane was blocked with 5% defatted milk and incubated with primary antibody (HIF-1α, 1:1,000; Abcam) at 4°C overnight and then secondary antibody (1:2000; ZSGB-BIO) at room temperature for 1 h. Finally, the protein bands were visualized with a super chemiluminescence detection reagent kit (Thermo Fisher Scientific).

### Reactive oxygen species assay (ROS)

ROS were detected using a reactive oxygen species assay kit (Beyotime Biotechnology, Shanghai, China). According to the instructions, cells grown on slices were incubated with serum-free medium containing 2′,7′-dichlorofluorescein-diacetate (DCFH-DA, 10 μM) for 20 min at 37°C. Then the cells were washed with PBS three times and examined under a fluorescence microscope.

### Statistical analysis

All experiments in this study were repeated at least three times. One-way analysis of variance and Student-Newman-Keuls tests were performed using GraphPad Prism 6 (GraphPad Software, La Jolla, CA, USA). The data are presented as means ± SD, and *P* < 0.05 was considered significant.

## Results

### LLLT promotes angiogenesis and regeneration of collagen fibers and bone tissue in BCP /BMSCs grafts in vivo

**Fig. 1:**
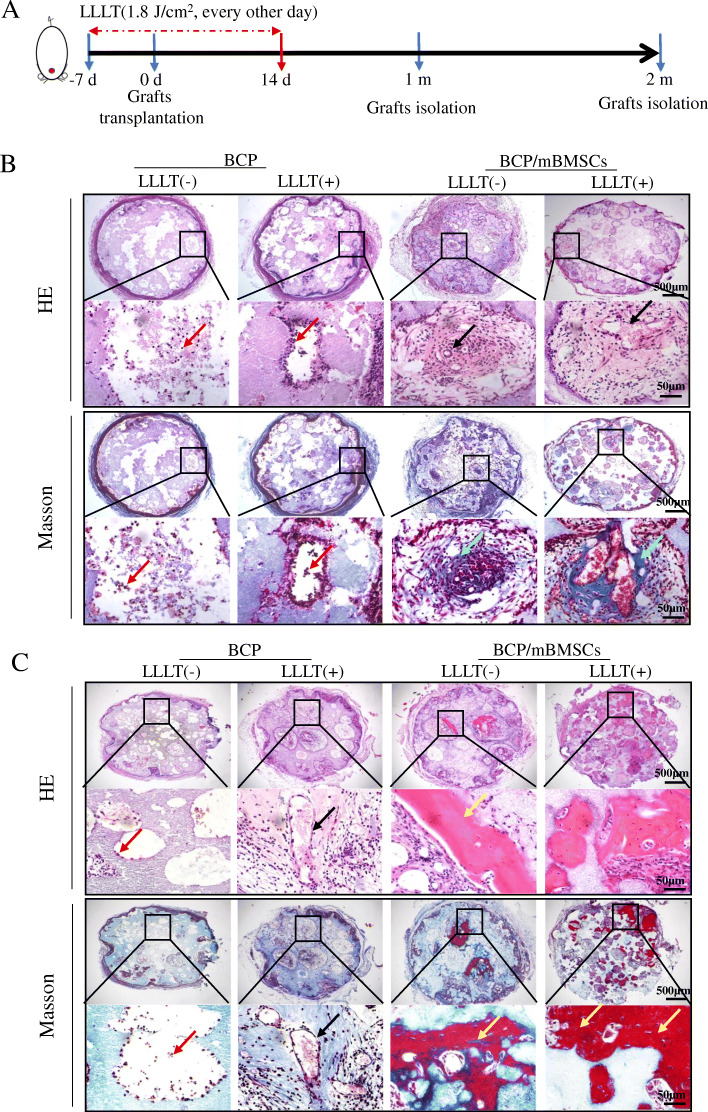
LLLT promoting the angiogenesis, collagen fiber formation, and mineralization in the mBMSC/BCP complex grafts. **A** Schematic representation of experimental design in vivo. The mice were divided into four groups: BCP group, BCP + LLLT group, mBMSC/BCP group, and mBMSC/BCP + LLLT group. The surgical area of mice undergoing LLLT (dose 1.8 J/cm^2^, 808 nm) every other day from 7 days before operation to 14 days after operation. One or 2 months after operation, 5 mice in each group were euthanized, and the implanted grafts were isolated for histological staining. **B** Representative images of the graft sections stained with H&E and Masson’s trichrome at 1 month after transplantation. Boxed areas in upper panel (scale bar, 500 μm) were shown in lower panel at higher magnification (scale bar, 50 μm). The red arrows marked the cells immersed in the pores of grafts, and the black arrows show the new blood vessels in the pores of grafts. **C** Representative images of the graft sections stained with H&E and Masson’s trichrome at 2 months after transplantation. The lower panel (scale bar, 50 μm) showing the magnification in the black boxes in upper panel (scale bar, 500 μm). The black arrows showing the new blood vessels in the pores of grafts, and the yellow arrows showing the new bone tissue in the pores of grafts

In order to investigate the effect of LLLT on bone regeneration and angiogenesis, we first established a model of C57BL/6 mice implanted with BCP or mBMSC/BCP complex under the skull periosteum (Fig. 1A, Additional file 2: Figure S1). Our in vitro cell seeding experiment showed that the BCP scaffolds supported the activity and attachment of mBMSCs (Additional file 2: Figure S1A, B). One month after implantation, the grafts were integrated with the skulls of the mice (Additional file 2: Figure S1C). The autologous mouse cells gradually grew into the pores of the grafts; less mineralized tissue was formed in mice implanted with BCP alone with or without LLLT, and H&E staining showed that ingrown cells were only visible at the edge of the BCP graft, while the numbers of cells and vascular-like structures in BCP+LLLT grafts were greater than in BCP alone grafts due to the effect of the laser enhancing cell angiogenesis. Compared with the BCP groups, more neovascularization and mineralized tissue formation were observed in mice implanted with BCP/mBMSC grafts, and LLLT further enhanced blood vessel formation and mineralization in these grafts (Fig. 1B). Masson’s trichrome staining showed that there was a large amount of collagen (blue-stained) and neovascularization in BCP/mBMSC grafts, especially in BCP/mBMSC+LLLT grafts (Fig. 1B). Two months after implantation, there were more vascular-like collagen structures in the BCP grafts treated with LLLT than the BCP alone grafts, but there was still no mineralized tissue, while there was not only more neovascularization and collagen fiber formation, but also some new bone tissue in the BCP/mBMSC+LLLT grafts, and the new bone tissue in the BCP/mBMSC+LLLT group exhibited a better degree of mineralization than that in the BCP+BMSC group (Fig. 1C). These results suggested that LLLT not only promoted known endothelial cell migration, angiogenesis, and BMSC differentiation, but also implied that LLLT may promote bone regeneration by enhancing the crosstalk between endothelial cells and BMSCs.

### LLLT promotes type H vessel formation in grafts

**Fig. 2:**
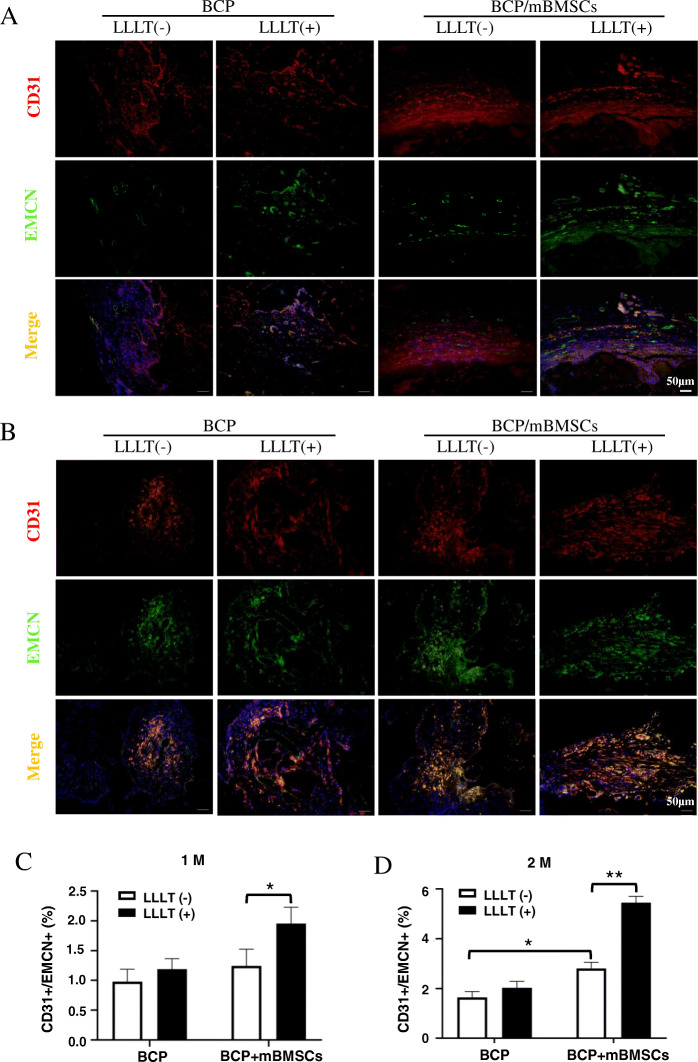
LLLT promoting the formation of type H vessels in BCP/mBMSCs complex grafts. **A**, **B** Double immunofluorescence staining was used to detect the CD31 and EMCN double-positive endothelial cells (type H vessels) in the grafts at 1 month and 2 months after transplantation. **C**, **D** The percentage of CD31 and EMCN double-positive endothelial cells was statistically analyzed in grafts 1 month and 2 months after transplantation, respectively. The yellow color represents the CD31 and EMCN double-positive cells. Scale bar, 50 μm (*P < 0.05, **P < 0.01)

Blood vessels not only provide necessary nutrition, oxygen, growth factors, and hormones for bone tissue, but also play an important role in the regulation of bone formation [28]. Recent studies have revealed that type H vessels (high expression of CD31 and EMCN) play a promoting role in the process of “angiogenesis and osteogenesis coupling” [11]. To test whether the effect of LLLT on promoting bone regeneration by grafts was related to type H vessels, we used immunofluorescence double staining to detect the expression levels of CD31 and EMCN in different graft groups. We found that 1 month after implantation, there was only a small number of type H vessels in the BCP alone group. LLLT had no significant effect on the formation of type H vessels in the BCP alone group, while LLLT increased formation of type H vessels in the BCP/mBMSC group (*P* < 0.05, Fig. 2A, C). Two months after implantation, the number of type H vessels was increased further compared with 1 month (Fig. 2B). The number of type H vessels in the BCP/mBMSC group was significantly higher than that in the BCP group, and LLLT further increased the number of type H vessels in the BCP/mBMSC grafts (*P* < 0.05, Fig. 2B, D). These results indicated that the transplanted stem cells contributed to host angiogenesis and type H vessel formation in BCP/mBMSC grafts during bone regeneration, while LLLT further enhanced angiogenesis, especially the formation of type H vessels, suggesting that LLLT might promote the coupling of angiogenesis and osteogenesis.

**Fig. 3:**
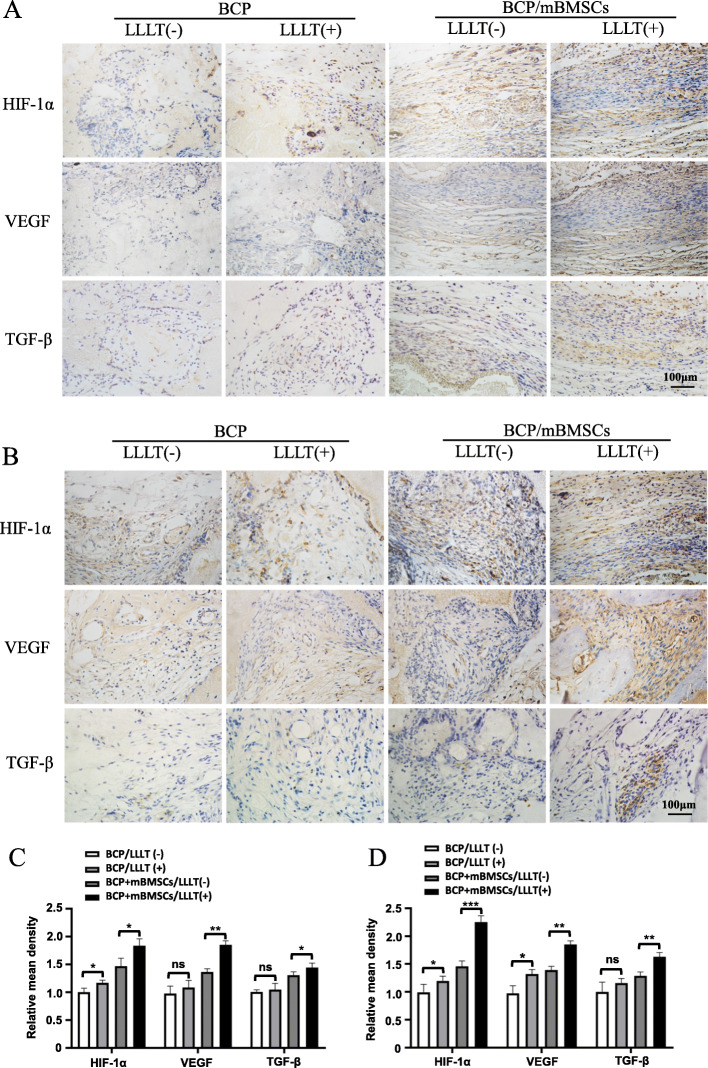
LLLT enhancing the expression of angiogenesis related factors in BCP/mBMSCs complex grafts. **A**, **B** Immunohistochemical staining was used to detect the expression of HIF-1 α, VEGF and TGF-β in grafts 1 month (**A**) and 2 months (**B**) after transplantation. **C**, **D** The quantitative analysis of HIF-1 α, VEGF, and TGF-β expression in grafts 1 month (**C**) and 2 months after transplantation using ImageJ software. Scale bar, 100 μm (ns, not statistically significant, *P < 0.05, **P< 0.01, ***P < 0.001)

Studies have shown that LLLT can promote angiogenesis and osteogenic differentiation of stem cells through the TGF-β pathway, and HIF-1α is an essential factor in the regulation of type H vessel formation. Next, we investigated the expression of those related factors by immunohistochemistry. The results showed that 1 month after implantation, LLLT increased the expression of HIF-1α in both BCP and BCP/mBMSC grafts, while it also increased the expression of VEGF and TGF-β in BCP/mBMSC grafts (Fig. 3A, C). Two months after implantation, LLLT induced the expression of HIF-1α and VEGF in both BCP and BCP/mBMSC grafts, but increased the expression of TGF-β only in BCP/mBMSC grafts (*P* < 0.05, Fig. 3B, D). These results were consistent with the histological features described above. It also showed that LLLT significantly increased the expression of HIF-1α (*P* < 0.001), and the expressions of VEGF and TGF-β (*P* < 0.01). Collectively, the above results indicated that LLLT enhanced the expression of angiogenesis-related factors, especially VEGF and HIF-1α, which further implied that LLLT might enhance bone regeneration by coupling angiogenesis and osteogenesis.

### LLLT promotes angiogenesis and osteogenic differentiation in a co-culture system of hBMSCs and HUVECs in vitro

To further explore whether LLLT plays a synergistic role in promoting the coupling of angiogenesis and osteogenesis, we established a co-culture system of endothelial cells and bone marrow mesenchymal cells in vitro to test the effects of LLLT on tube formation and osteogenic differentiation potential of the cells. We selected the 4.5 J/cm^2^ dose of LLLT for subsequent experiments based on preliminary cell proliferation and ROS assay experiments (Additional file 3: Figure S2).

**Fig. 4:**
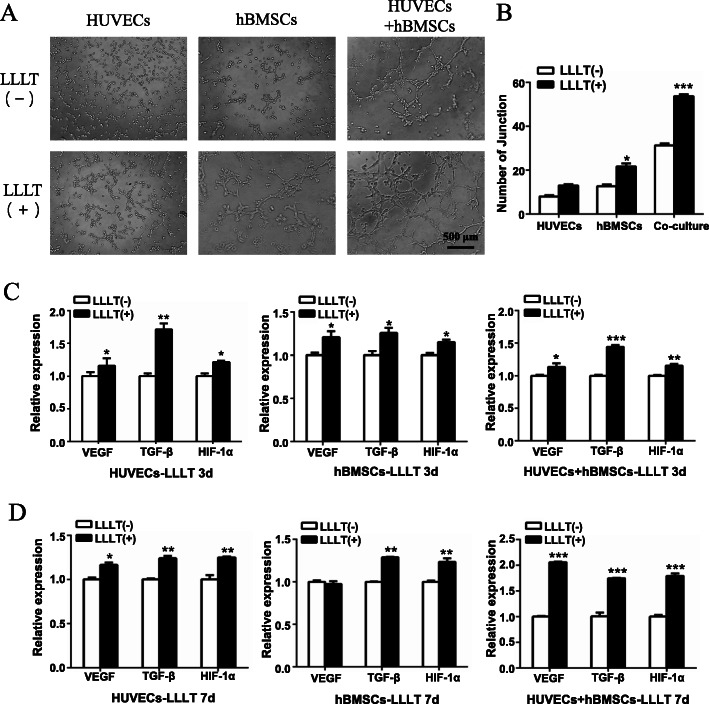
LLLT increasing angiogenesis in co-culture system of hBMSCs and HUVECs. **A** The representative tube formation images of HUVECs, hBMSCs, co-cultured HUVECs, and hBMSCs with or without LLLT (4.5 J/cm^2^ every day), scale bar, 500 μm. **B** Quantification of the junction number using Image J software. **C**, **D** The expression of the angiogenesis-related genes (*VEGF*, *TGF-β*, and *HIF-1α*) was measured by RT-qPCR at 3 days (**C**) and 7 days (**D**) under osteogenic induction (*P < 0.05, **P < 0.01, ***P < 0.001)

First, a tube formation experiment was carried out to explore the effects of LLLT on the angiogenic potential of hBMSCs, HUVECs, and co-cultured cells. Our results verified that LLLT promoted the formation of tube-like structures in all three types of cultures, but especially the co-cultured hBMSCs and HUVECs (Fig. 4A). Quantitative analysis of the junction number further confirmed that LLLT significantly enhanced the tube-forming ability of co-cultured cells (Fig. 4B), suggesting that crosstalk between hBMSCs and HUVECs in the co-culture system enhanced tube formation ability.

Next, we measured the effect of LLLT on angiogenesis- and osteogenesis-related gene expression under osteogenic conditions, including expression of VEGF, TGF-β, and HIF-1α. The results showed that LLLT (3 days) upregulated VEGF, TGF-β, and HIF-1α expression in HUVECs, hBMSCs, and co-cultures (Fig. 4C). After 7 days, LLLT enhanced the expression of TGF-β and HIF-1α in HUVECs, hBMSCs, and co-cultured cells, but only increased the expression of VEGF in both HUVECs and co-culture groups. However in the co-culture group, the expression of VEGF, TGF-β, and HIF-1α all increased sharply, and the effect was enhanced in the co-culture system (Fig. 4D). HIF-1α is an important transcription factor related to angiogenesis, which is closely associated with the formation of type H vessels [12]. We found that the expression of HIF-1α in the LLLT groups was higher than that in the non-LLLT groups, and the difference was most significant in the co-culture system (*P* < 0.001, Fig. 4D). TGF-β is a key factor related to angiogenesis and osteogenesis. Studies have shown that LLLT activates latent TGF-β by regulation the level of ROS, thereby promoting osteogenic differentiation of stem cells [24]. Our results showed that the expression pattern of TGF-β in cultures treated by LLLT was consistent with that of HIF-1α. In particular, LLLT increased the expression of TGF-β in vascular endothelial cells in the early stage and also enhanced TGF-β expression significantly more in co-cultures compared with HUVECs or hBMSCs alone (*P* < 0.001, Fig. 4D). All the above results implied that LLLT could further enhance promotion of angiogenesis in the co-culture system, due to the crosstalk between vascular endothelial cells and bone marrow mesenchymal stem cells.

**Fig. 5:**
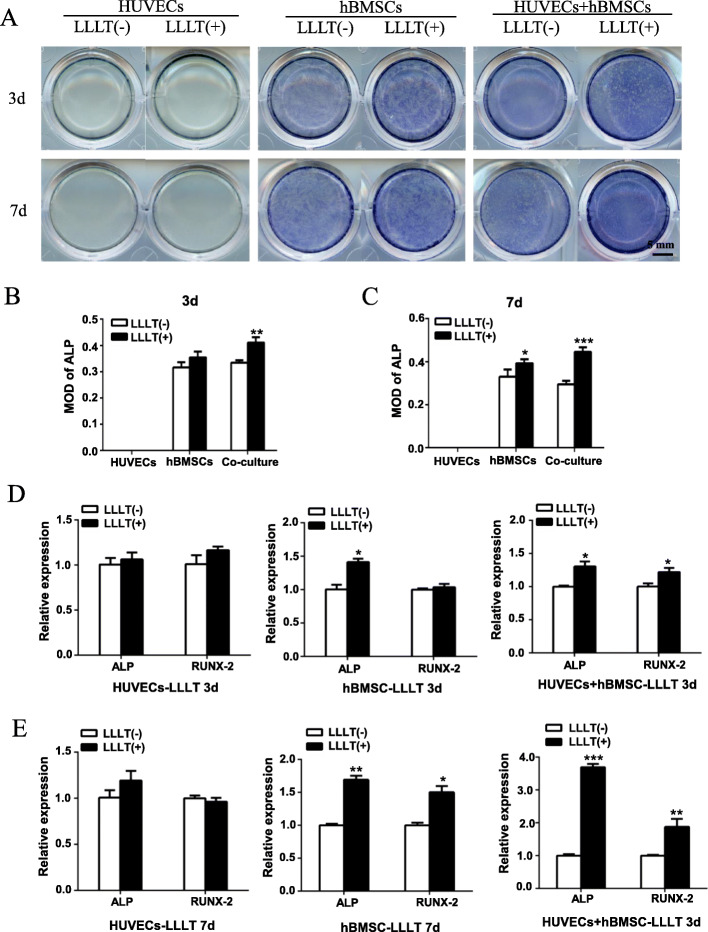
LLLT enhancing osteogenic potential of the co-cultured hBMSCs and HUVECs during early stage of osteogenic induction. **A** HUVECs, hBMSCs, the co-cultured hBMSCs, and HUVECs were stained with ALP at 3 days and 7 days after osteogenic induction to assess their osteogenic ability in the early stage. **B**, **C** Quantification and analysis of the ALP staining by mean optical density (MOD) at 3 days and 7 days. **D**, **E** The expression level of the osteogenesis-related genes (*ALP, RUNX-2*) was measured by RT-qPCR at 3 days (**D**) and 7 days (**E**) after osteogenic induction (*p < 0.05, **p < 0.01, ***p < 0.001)

Vascularization is a crucial process during the healing and regeneration of bone [28]. Previous studies have shown that co-culture of endothelial cells and mesenchymal stem cells promotes bone differentiation of stem cells [29]. To investigate the effect of LLLT on osteogenic differentiation in the co-culture system of HUVECs and hBMSCs, ALP staining was performed in HUVECs, hBMSCs, and co-cultures at 3 days and 7 days to evaluate early stage induction of osteogenesis. The results showed that LLLT had no effect on the osteogenic potential of HUVECs (Fig. 5A–C). ALP staining and quantitative analysis showed that in hBMSCs and in co-cultures after 3 days or 7 days of osteogenic induction, the activity of ALP increased gradually and was further enhanced by LLLT, especially in the co-culture system (Fig. 5A–C). RT-qPCR results showed that LLLT increased the expression of ALP and RUNX2 after 3 days of osteogenic induction in the co-culture group, and further enhanced the expression of both after 7 days of osteogenic induction, while LLLT only increased the expression of RUNX2 after 7 days of osteogenic induction in cultures of hBMSCs alone (Fig. 5D, E). This result is consistent with the results of ALP staining.

**Fig. 6:**
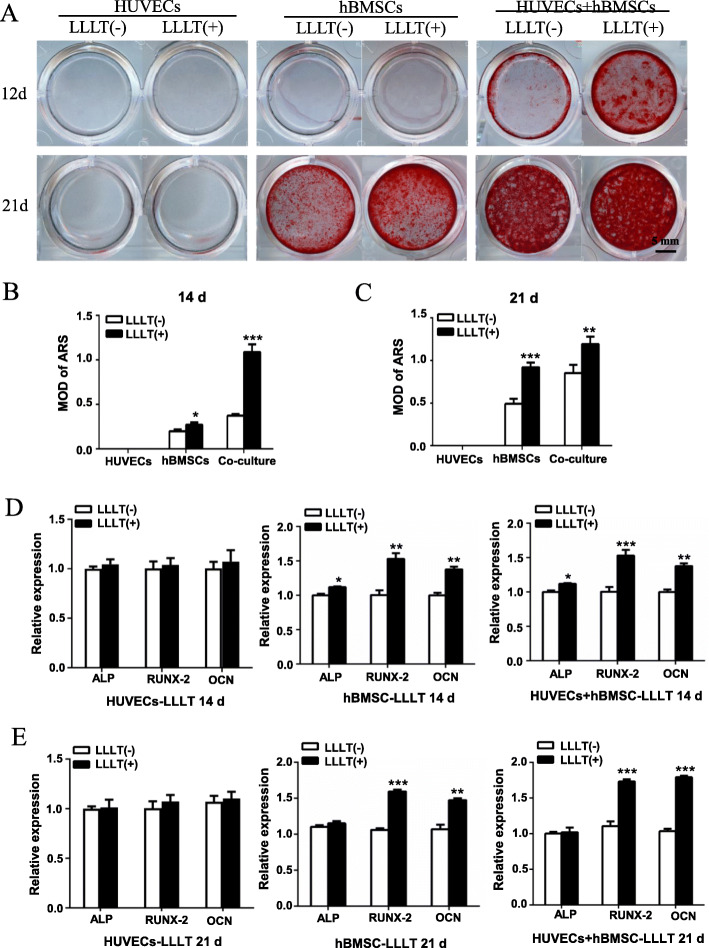
LLLT promoting osteogenic potential of the co-cultured hBMSCs and HUVECs in late stage of osteogenic induction. **A** HUVECs, hBMSCs, and the co-cultured hBMSCs and HUVECs were stained with Alizarin Red S (ARS) at 14 days and 21 days after osteogenic induction. **B**, **C** Quantification of the ARS staining by MOD at 14 days and 21 days. **D**, **E** The expression of the osteogenic genes (*ALP, RUNX-2, OCN*) was measured by RT-qPCR at 14 days (**D**) and 21 days (**E**) after osteogenic differentiation (ns, not statistically significant, *P < 0.05, **P < 0.01, ***P < 0.001)

Next, we carried out ARS staining after 14 days and 21 days to evaluate late-stage osteogenic induction. For HUVECs, there was still no osteogenic differentiation or calcification as revealed by ARS staining. In the co-culture system, obvious calcium nodules were visible after 14 days of osteogenic induction, while few were formed in the hBMSCs alone group; at 21 days, calcium nodules appeared in the hBMSCs group, but were significantly fewer than in the co-culture group. In contrast, after 14 or 21 days with LLLT treatment, the number of calcium nodules deposited by BMSCs in both the co-culture group and the hBMSCs alone group was significantly greater than that in the non-LLLT treatment group, and the effect of LLLT on the co-culture group was the strongest (Fig. 6A–C). The result of RT-qPCR showed that after 14 and 21 days of LLLT irradiation, the expression of ALP, as an early stage marker of osteogenesis, in the LLLT(+) group was slightly higher than that in the LLLT(−) group, while the expression of Runx2 and OCN in the LLLT(+) group was significantly higher than that in the LLLT(−) group, especially in co-cultures (Fig. 6D, E). These results indicated that LLLT promoted both the early and late stages of osteogenic differentiation of BMSCs, and this effect was enhanced in the co-culture system, suggesting that LLLT may further promote the synergistic effect by coupling osteogenesis and angiogenesis.

### LLLT promotes the coupling of angiogenesis and osteogenesis through the ROS/HIF-1α axis

**Fig. 7:**
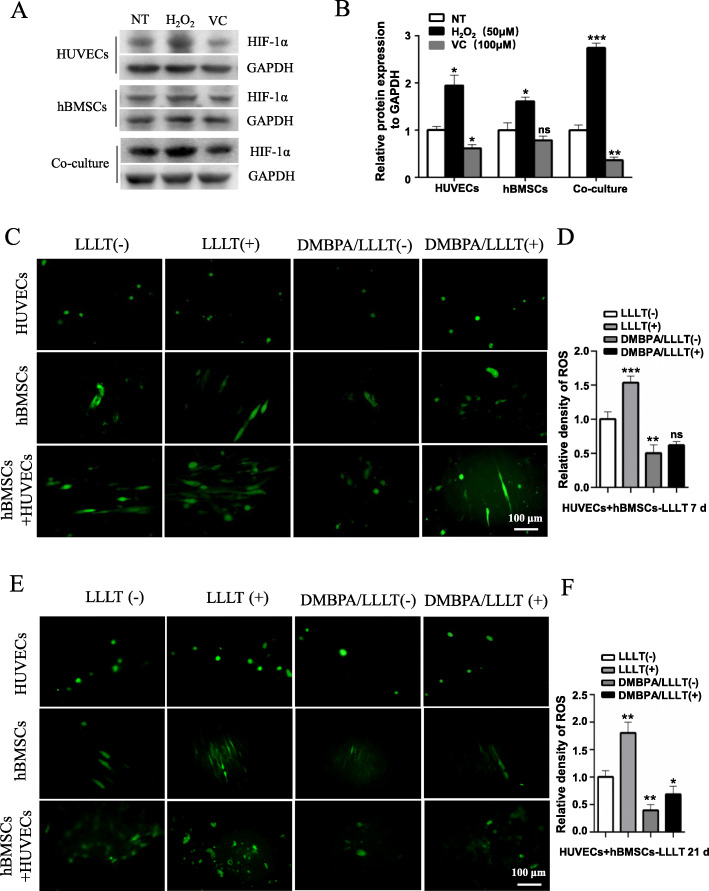
LLLT activating ROS/HIF-α signaling pathway in co-culture system. **A** In HUVECs, hBMSCs, and co-cultured HUVECs and hBMSCs, H_2_O_2_ (50 μM, 30min) or vitamin C (100 μM, 24h) was added, respectively. The expression of HIF-1α was detected by Western blot. **B** Western blot and quantification of HIF-1α. **C** The expression of ROS (green) was detected by ROS assay after 7 days of LLLT (4.5 J/cm^2^, every day) in HUVECs, hBMSCs, and co-cultured HUVECs and hBMSCs, scale bar, 100 μm. **D** The quantification of ROS by mean optical density after 7 days of LLLT. **E** The expression of ROS (green) was detected after 21 days of LLLT. **F** The quantification of ROS after 21 days of LLLT, scale bar, 100 μm (ns, not statistically significant , *p < 0.05, **P < 0.01, ***P < 0.001)

HIF-1α is a transcription factor that mediates cellular activity in response to changes in oxygen levels, and controls physiological and pathological neo-angiogenesis. Endothelial HIF-1α is a significant promoter of type H vessel formation, which plays a crucial role in bone formation and regeneration [12]. Previous studies have shown that the tricarboxylic acid (TCA) cycle along with the electron transport chain (ETC) increases ROS production in mitochondria and further contributes to HIF-1α stabilization [30]. Recent research has proved that LLLT promotes bone differentiation of stem cells by activating latent TGF-β through increasing internal ROS [24]. Our in vivo experimental results showed that the protein level of HIF-1α was significantly increased by LLLT. In order to further explore the mechanism, we first regulated the level of ROS in the co-culture system and observed the protein level of HIF-1α. The results showed that the protein level of HIF-1α was upregulated significantly when oxidant H_2_O_2_ was added to the co-culture system, and downregulated obviously when antioxidant vitamin C (VC) was added to the co-culture system (Fig. 7A, B). Next, we added Dimethyl-bisphenol A (DMBPA), an inhibitor of HIF-1α that promotes degradation of HIF-1α protein, into the co-culture system, and then analyzed the level of ROS. The results showed that LLLT effectively increased the production of ROS in the co-culture system (Fig. 7C, D). Importantly, when DMBPA was added to the co-culture system, the production of ROS decreased significantly, while after LLLT treatment in the DMBPA group, ROS production was not increased obviously, suggesting that LLLT might promote osteogenesis and angiogenesis in co-cultures of hBMSCs and HUVECs through the ROS/HIF-1α signaling pathway.

**Fig. 8:**
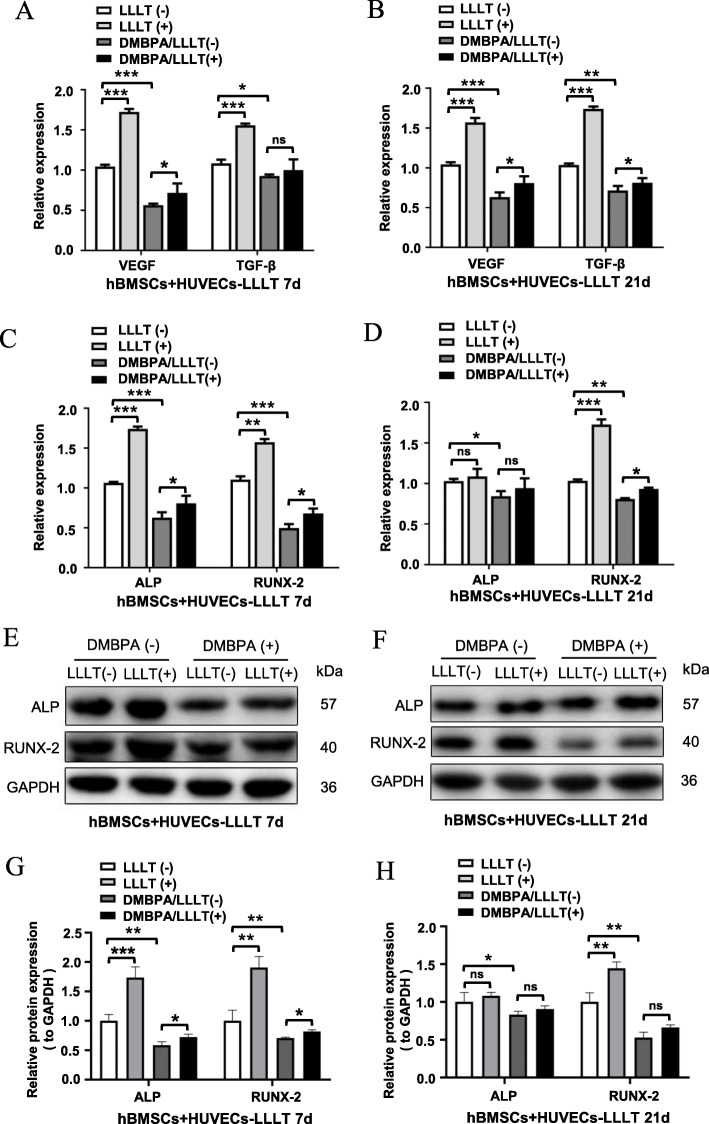
LLLT promoting coupling angiogenesis and osteogenesis through ROS/HIF-1α pathway in the co-culture system. **A** The HIF-1α inhibitor DMBPA (50 μM) was added into the co-culture system. After 7 days of LLLT treatment, the angiogenesis related factors (VEGF and TGF-β) were detected by RT-qPCR. **B** After 21 days of LLLT treatment, the expression of angiogenesis-related factors (VEGF and TGF-β) was detected by RT-qPCR; **C** Detecting the mRNA expression of osteogenic-related factors (ALP and RUNX-2) by RT-qPCR at 7 days. **D** Detecting the osteogenesis-related factors (ALP and RUNX-2) by RT-qPCR at 21 days. **E**, **G** Detecting and analyzing the expression of osteogenesis-related protein (ALP and RUNX-2) by western blot at 7 days. **F**, **H** Detecting and analyzing the expression of osteogenesis-related protein (ALP and RUNX-2) by western blot at 21 days (ns, not statistically significant, *P< 0.05, **P< 0.01, ***P < 0.001)

To better understand the association between ROS and HIF-1α, we then analyzed expression of the angiogenesis-related factors VEGF and TGF-β by RT-qPCR, and the osteogenic-related factors ALP and Runx2 by RT-qPCR and western blot. The results showed that DMBPA significantly inhibited the expression of VEGF and TGF-β in co-cultures after 7 and 21 days of osteogenic induction. However, compared with the DMBPA group, the expression of VEGF and TGF-β was not increased obviously in the DMBPA + LLLT group (Fig. 8A, B). The mRNA expression and protein level trend of ALP and Runx2 was consistent with that of VEGF and TGF-β (Fig. 8C–H). These results further indicated that the ROS/HIF-1α signal pathway played a critical role in the process via which LLLT exerted its synergistic effect of coupling angiogenesis and osteogenesis.

## Discussion

It is crucial for tissue-engineered bone to form vascularized bone as early as possible and promote the differentiation of stem cells into osteoblasts. Crosstalk between MSCs and the vascular network is the basis of bone formation, remodeling, and healing processes, and the recruitment and migration of endothelial cells are critical to these processes [31–33]. Recent studies have confirmed that type H blood vessels and bone formation are closely related [34]. Low energy laser therapy has many biological effects. In recent years, many studies have shown that LLLT promotes the differentiation of bone marrow mesenchymal stem cells and promotes endothelial cells angiogenesis in vitro [16, 35, 36]. Meanwhile in vivo studies have confirmed that LLLT promotes angiogenesis, osteogenic differentiation of stem cells, and fracture healing [37, 38]. However, whether LLLT is coupled with blood vessel formation, and bone formation is related to bone repair and regeneration, is not very clear. Here, our results showed that LLLT enhanced vascularized bone regeneration by coupling angiogenesis and osteogenesis. ROS/HIF-1α was necessary for formation of type H vessels and osteogenic differentiation of mesenchymal stem cells induced by LLLT. LLLT triggered a ROS-dependent increase of HIF-1α and TGF-β, resulting in crosstalk between angiogenesis and osteogenesis. As ROS also was a target of HIF-1α, there may be a positive feedback loop between ROS and HIF-1α, which further amplified HIF-1α induction via the LLLT-mediated ROS increase.

LLLT is a general name to refer to laser therapy based on photobiomodulation and has been widely used for over 50 years. Helium-neon (He-Ne) and gallium-aluminum-arsenide (Ga-Al-As) lasers are most commonly used for LLLT. LLLT has been discovered to have a variety of biological effects such as relief of pain and inflammation, promoting wound healing, callus growth, nerve repair, improving immunity and blood circulation, and promoting metabolism by regulating the pathophysiological state of the human immune, nervous, blood circulatory, and tissue metabolic systems. The biological effects of LLLT at the cellular and molecular levels could include cellular viability, proliferation, and DNA integrity and repair [39]. LLLT has gradually been widely adopted clinically. In recent years, LLLT has also been used for wound healing, fracture healing, repair of calvaria bone defects, and dental tissue regeneration due to its potential to promote tissue regeneration [22, 38, 40, 41]. Although the biological mechanisms underlying the effects are not fully understood, it is generally believed that the regenerative potential of LLLT may be related to its promotion of cell proliferation, angiogenesis, and differentiation of stem cells, among other effects [39]. Our results indicated that LLLT played a synergistic role in the vascularization and osteogenic differentiation of stem cells in grafts.

The effects of LLLT on proliferation, tube formation, and differentiation of cultured cells vary according to the applied wavelengths, output power, and energy density [26, 42]. Most LLLT are conducted using wavelengths of 600–1100 nm, and an output power of 1–500 mW in an energy density of 0.1–100 J/cm^2^ [43, 44]. Soleimani et al. discovered that LLLT (810 nm) in the energy density range of 2–6 J/cm^2^ promoted the proliferation of BMSCs and their differentiation into neurons and osteoblasts [45]. Wang et al. confirmed that LLLT (1064 nm) significantly promotes the proliferation of BMSCs at an energy density between 2 and 4 J/cm^2^ as well as osteogenic differentiation of BMSCs at an energy density of 2–4 J/cm^2^ and has no effect on the proliferation and osteogenic differentiation of BMSCs at 8 J/cm^2^ or 16 J/cm^2^, respectively [26]. Another study showed that LLLT (650 nm) promotes the proliferation, migration, and tube formation of HUVECs in a dose- and time-dependent manner at energy densities of 1, 2, or 4 J/cm^2^ [35]. Our results showed that LLLT (Ga-Al-As, 808 nm) at 4.5 J/cm^2^ has a better effect on promoting proliferation of HUVECs, BMSCs, and MSCs co-cultured with HUVECs than 1.5 J/cm^2^ or 7.5 J/cm^2^. Our subsequent research in vitro also confirmed that LLLT at 4.5 J/cm^2^ enhanced tube formation of the above three types of cells and osteogenic differentiation of BMSCs and BMSCs co-cultured with HUVECs. Our in vivo experiment showed that 40 MW for 3 min (1.8 J/cm^2^) had better promoting effect on wound healing and osteogenesis in vivo. However, when using the higher dose of LLLT, the mouse skin was damaged, which may be because the dark skin of the mouse absorbs too much energy to cause thermal damage. Therefore, the laser dose of 4.5 J/cm^2^ was used for in vitro experiment and 1.8 J/cm^2^ for in vivo experiments. Further work is still needed to understand the specific variation in the future.

Many studies have confirmed that the co-culture of endothelial cells and mesenchymal stem cells can promote osteogenic differentiation. Further studies have shown that the ratio of both ECs and MSCs also affects osteogenic differentiation (47), but the results vary, which may be related to the cells used. We chose the current co-culture ratio because the endothelial cell line used in our co-culture system grows faster than stem cells, which affects the osteogenic differentiation of stem cells under the condition of 1:1 ratio. We tested co-culture conditions with different cell ratios and screened optima cell ratio. Our results showed that the proportion of HUVECs was almost 50% in hBMSCs and HUVECs co-cultured at 5:1 with enhanced ALP staining. Further work is required needed to understand the specific variation in the future.

Mechanistic studies showed that LLLT promotes the proliferation, migration, and angiogenesis of HUVECs via activation of the PI3K/Akt signaling pathway accompanied with increases in the levels of angiogenesis-related genes (HIF-1α, eNOS, and VEGFA) [35]. LLLT also enhances the osteogenic differentiation of stem cells by inducing ROS which activates latent TGF-β1 [24] or stimulates the BMP/Smad signaling pathway [44]. TGF-β is secreted as a latent complex, and its activation is a key step in its physiological function. Several methods of latent TGF-β activation have been described, including extreme pH, heat, ultrasound, integrin binding, ionizing radiation, and proteases, such as thrombospondin-1. These TGF-β triggers have various degrees of attractiveness for clinical application because of practical and safety concerns. Our results showed that LLLT might activate endogenous TGF-β for osteogenesis.

Previous results showed that the interaction of ECs and stem cells promotes osteogenic differentiation and vascularized bone regeneration and also enhances angiogenic factor expression [46–48]. Moreover, blood vessel growth is associated with bone formation, especially the specialized type H vessels. Recent studies have identified the CD31^hi^EMCN^hi^ vascular endothelium that positively regulates bone formation, bone maturation, and regeneration. Studies have shown that type H vessel coupling bone formation is involved in a variety of factors, including HIF-1α, Notch, VEGF, platelet-derived growth factor type BB (PDGF-BB), slit guidance ligand 3 (SLIT3) [12, 34, 49, 50]. Given that LLLT has the dual effect of promoting tube formation by ECs and the osteogenic differentiation of stem cells, and our results also confirmed that LLLT could further enhance tube formation and osteogenic differentiation in a co-culture cell system, we hypothesized that LLLT may exert those effects through coupling of angiogenesis to osteogenesis. Our results in vivo confirmed this hypothesis, showing that LLLT induced more type H blood vessels. It is known that HIF-1α is crucial in maintaining formation of type H blood vessels and osteoprogenitors [34, 49, 50], so we measured HIF-1α expression. Our results in vivo and in vitro showed that LLLT did increase the protein level of HIF-1α, accompanied by an increase of angiogenesis-related VEGF and TGF-β1 expression. Promoting degradation of HIF-1α protein resulted in a significant decrease in the expression of LLLT-induced genes related to angiogenesis and osteogenesis. Previous study confirmed that LLLT induced ROS to activate latent TGF-β [24]; thus, we further investigated whether LLLT induced ROS production as well as enhancing HIF-1α function, and our results are in agreement with a recent study performed in monocytes and macrophages demonstrating that ROS are strong inducers of HIF-1α which can be induced via ROS-mediated inhibition of PHDs [51, 52]. ROS can promote osteogenic differentiation only in a suitable window, and higher ROS will damage osteogenic differentiation. Our study showed that the level of ROS was upregulated with the increase of LLLT dose. When the level of ROS was increased to 20%, the osteogenic ability of the co-culture system was the best. When ROS production in the co-culture system is increased or decreased, HIF-1α is also decreased or increased accordingly. Interestingly, when HIF-1α was inhibited, ROS decreased accordingly. A possible explanation was that there was a feedback loop between HIF-1α and ROS, which requires further study.

Our results suggested that there were several mechanisms via which LLLT promoted fracture healing and bone regeneration in clinical practice. On the one hand, LLLT promoted angiogenesis through angiogenesis-related factors to enhance local nutrient supply. On the other hand, LLLT-induced ROS activated TGF-β to promote the osteogenic differentiation of stem cells and produce more mineralized bone. Finally, LLLT-induced ROS enhanced the stability of HIF-1α, which resulted in type H blood vessels to couple angiogenesis and osteogenesis. However, some studies have conflicting results for the effects of LLLT, which may be a result of inconsistent treatment protocols that require further study.

In short, our study provided an explanation of the mechanism via which LLLT accelerated fracture healing and repair of bone defects, and further research will contribute to expand the application of LLLT in regenerative medicine.

## Conclusion

The results of this study supported the concept that LLLT enhanced vascularized bone regeneration by coupling angiogenesis and osteogenesis. ROS/HIF-1α may be the key to formation of type H vessels and enhancement of osteogenic differentiation of MSCs. The enhanced growth of blood vessels further promoted bone repair and regeneration. Our study provided new insights into understanding the role of LLLT in fracture healing and tissue engineering strategies.

## Supplementary Information

**Additional file 1.** Table S1 Primer sequence of the target genes.

**Additional file 2.** Figure S1. Construction of mBMSCs/BCP bone tissue grafts. A mBMSCs labeled with GFP (green fluorescence) were co-cultured with BCP for 7 days. B left panel: immunofluorescence staining showing the mBMSCs (green) stained with DAPI (blue) in BCP 7 days after co-culture, scale bar, 100 μm; right panel showed the magnification in the box of left panel, scale bar, 50 μm. C The graft dissected from the skull periosteum of C57BL/6 mice at 1 month after operation, showing the grafts were integrated with the skull of mice, scale bar, 1 mm.

**Additional file 3.** Figure S2. LLLT promotes cell proliferation and ROS in the co-culture of hBMSCs and HUVECs. A Bright-field image of hBMSCs, HUVECs, co-culture of hBMSCs and HUVECs, scale bar, 100 μm. B, C LLLT on cell proliferation of co-culture of hBMSCs and HUVECs for 1-5 days was investigated by CCK8 assay, showing LLLT at the dose of 100 mW 3 min (4.5 J/cm2) was better than others. D The ROS assay and ALP staining was performed to detect the effects of LLLT on the ROS level and the osteogenic ability in co-culture system at 5 days. E Quantitative analysis of the effects of different doses of LLLT on the ROS level at 5 days in the co-culture system. To sum up, the effect of LLLT at the dose of 100 mW 3 min (4.5 J/cm2) was better than others.

## Data Availability

The datasets supporting the conclusions of this article are included within the article and its additional files.

## Abbreviations

ALP: Alkaline phosphatase
BCP: Biphasic calcium phosphate
BMSCs: Bone marrow mesenchymal stem cells
DAPI: 4′,6-Diamidino-2-phenylindole
EMCN: Endomucin
FBS: Fetal bovine serum
HE: Hematoxylin and eosin
HIF-1α: Hypoxia-inducible transcription factor 1α
LLLT: Low-level laser therapy
MSCs: Mesenchymal stem cells
OCN: Osteocalcin
PBS: Phosphate-buffered saline
ROS: Reactive oxygen species
Runx2: Runt-related transcription factor 2
TGF-β: Transforming growth factor-β
VEGF: Vascular endothelial growth factor
PHDs: Proline hydroxylases
