# Supplementary material for: Low level laser therapy promotes bone regeneration by coupling angiogenesis and osteogenesis
Source: Stem Cell Res Ther. 2021 Aug 3;12:432. doi: 10.1186/s13287-021-02493-5 (PMC8330075; doi:10.1186/s13287-021-02493-5)
Supplement: Supplementary file 1 — Additional file 1. Table S1 Primer sequence of the target genes. [file 13287_2021_2493_MOESM1_ESM.docx]

Table S1 Primer sequence of the target genes

| Primer | Forward primer | Reverse primer |
| --- | --- | --- |
| ALP | ACCATTCCCACGTCTTCACATTT | AGACATTCTCTCGTTCACCGCC |
| RUNX-2 | AGCAAGGTTCAACGATCTGAGAT | TTTGTGAAGACGGTTATGGTCAA |
| OCN | ATGAGAGCCCTCACACTCCT | GGATTGAGCTCACACACCTC |
| VEGF | AGGAGTACCCTGATGAGATCGAGTA | TGGTGAGGTTTGATCCGCATA |
| TGF-β | CGCCAGAGTGGTTATCTTTTG | CGGTAGTGAACCCGTTGATGT |
| HIF-1α | ACCGCTGAAACGCCAAAG | TCCATCGGAAGGACTAGGTGTCT |
| GAPDH | GTGAAGGTCGGAGTCAACG | TGAGGTCAATGAAGGGGTC |
