# Supplementary figures and images for: Low level laser therapy promotes bone regeneration by coupling angiogenesis and osteogenesis
Source: Stem Cell Res Ther. 2021 Aug 3;12:432. doi: 10.1186/s13287-021-02493-5 (PMC8330075; doi:10.1186/s13287-021-02493-5)

A

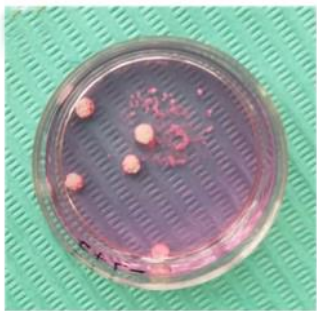

B

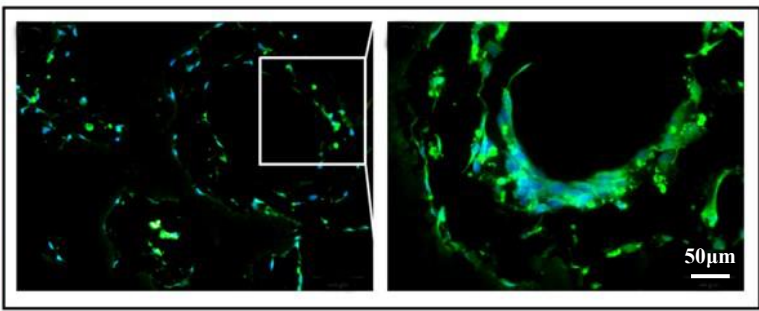

C

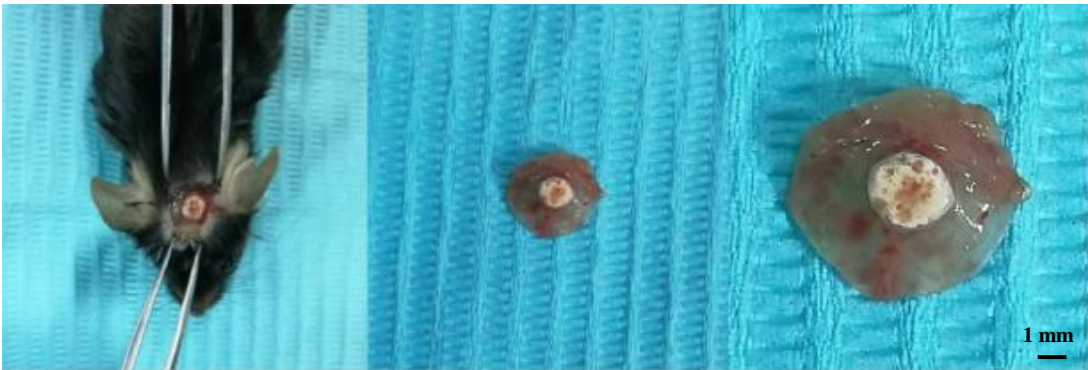

Supplement: Supplementary file 2 — Additional file 2. Figure S1. Construction of mBMSCs/BCP bone tissue grafts. A mBMSCs labeled with GFP (green fluorescence) were co-cultured with BCP for 7 days. B left panel: immunofluorescence staining showing the mBMSCs (green) stained with DAPI (blue) in BCP 7 days after co-culture, scale bar, 100 μm; right panel showed the magnification in the box of left panel, scale bar, 50 μm. C The graft dissected from the skull periosteum of C57BL/6 mice at 1 month after operation, showing the grafts were integrated with the skull of mice, scale bar, 1 mm. [file 13287_2021_2493_MOESM2_ESM.pdf]

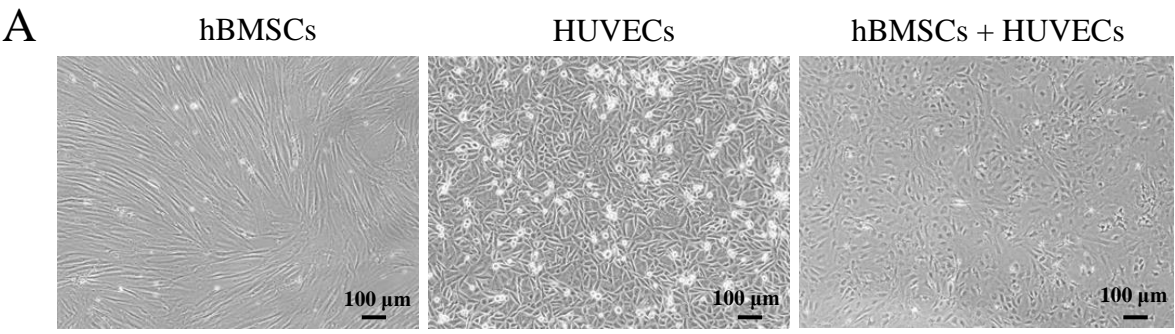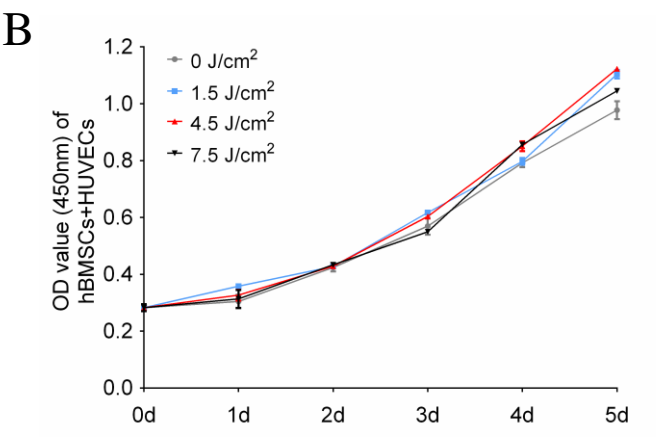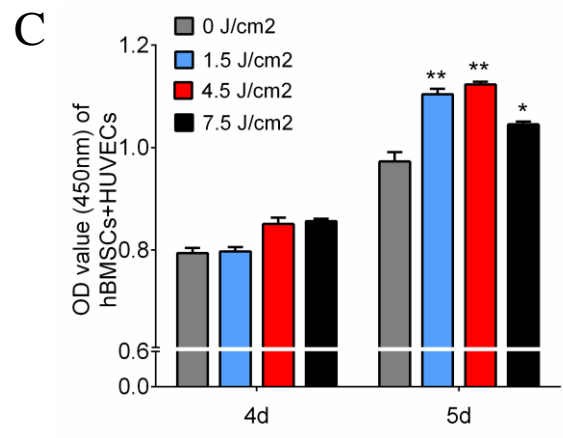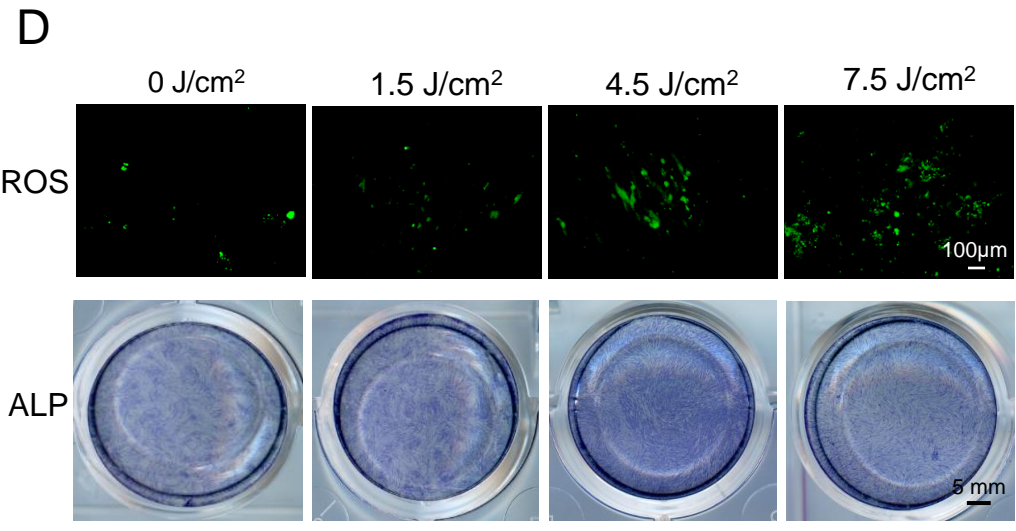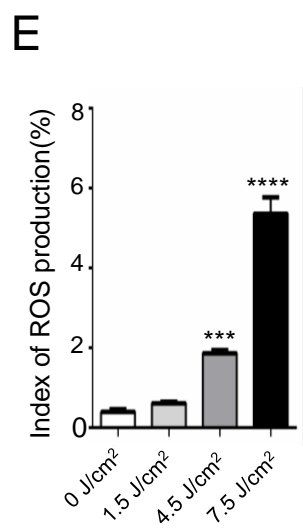

Supplement: Supplementary file 3 — Additional file 3. Figure S2. LLLT promotes cell proliferation and ROS in the co-culture of hBMSCs and HUVECs. A Bright-field image of hBMSCs, HUVECs, co-culture of hBMSCs and HUVECs, scale bar, 100 μm. B, C LLLT on cell proliferation of co-culture of hBMSCs and HUVECs for 1-5 days was investigated by CCK8 assay, showing LLLT at the dose of 100 mW 3 min (4.5 J/cm2) was better than others. D The ROS assay and ALP staining was performed to detect the effects of LLLT on the ROS level and the osteogenic ability in co-culture system at 5 days. E Quantitative analysis of the effects of different doses of LLLT on the ROS level at 5 days in the co-culture system. To sum up, the effect of LLLT at the dose of 100 mW 3 min (4.5 J/cm2) was better than others. [file 13287_2021_2493_MOESM3_ESM.pdf]
